# The effectiveness of low-level laser therapy and low-intensity pulsed ultrasound in reducing pain induced by orthodontic separation: a randomized controlled trial

**DOI:** 10.1186/s12903-024-03926-2

**Published:** 2024-02-02

**Authors:** Lama Mohammad Saffouh Al-Hanbali, Ahmad Sharafeddin Burhan, Mohammad Younis Hajeer, Fehmieh Rafik Nawaya

**Affiliations:** 1 Department of Orthodontics and Dentofacial Orthopedics, Faculty of Dental Medicine, Damascus University, Al-Mazzeh Street, Damascus, Syria; 2 Department of Pediatric Dentistry, Faculty of Dental Medicine, Syrian Private University, Daraa, Syria

**Keywords:** Pain, Discomfort, Orthodontic separation, Elastomeric separation, Low-level laser, Low-intensity pulsed ultrasound

## Abstract

**Background:**

The low-level laser therapy (LLLT) and low-intensity pulsed ultrasound (LIPUS) have been recently applied to control pain during orthodontic treatment.

**Objective:**

To evaluate and compare the effectiveness of LLLT and LIPUS in reducing pain induced by orthodontic separation.

**Study design:**

A single-blinded randomized controlled trial.

**Methods:**

One hundred and fifty patients were randomly assigned into three groups; LLLT group, LIPUS group, and control group. After 5 min from the separators’ placement, the first dose of the laser or the ultrasound was applied, the second dose was applied after 24 h, and the third dose was applied after 48 h on both maxillary and mandibular first molars. The exposure of laser was for 20 s at each point (maxillary and mandibular first molars), with an 810-nm aluminum-gallium-arsenide (AlGaAs) diode laser on continuous mode. The output power set at 150 mW, the energy density of 4 J/cm^2^, and a laser spot diameter of 7 mm were applied. Whereas the frequency of ultrasonic toothbrush was 1.6 MHz; and average output intensity was 0.2 W/cm^2^. The application was for 20 min (5 min on each first molar). The control group received the separators without another intervention. A Visual Analog Scale (VAS 100 mm) was used to assess pain intensity at several time intervals during the first four days after the separators’ placement.

**Results:**

A total of 145 patients were assessed. There was a significant difference in pain perception among the three groups after 5 min (*P* = .002). The maximum pain level was reached after 24 h. However, the laser group and the ultrasound group showed a statistically significant decrease in pain scores compared to the control group at all the assessment time points (*P* < .001). Whereas there was no difference between the laser group and the ultrasound group in reducing the pain scores (*P* > .05).

**Conclusions:**

The LLLT and the LIPUS effectively reduce the separation pain when applied in multiple doses without differences between them.

**Trial registration:**

This trial was registered with the German Clinical Trials Register (DRKS). (https://www.drks.de/drks_web/navigate.do?navigationId=trial.HTML&TRIAL_ID= DRKS00029991). Date of registration: 26/08/2022.

**Supplementary Information:**

The online version contains supplementary material available at 10.1186/s12903-024-03926-2.

## Background

Teeth movement causes many side effects during the orthodontic treatment, pain is the foremost expected side impact, which is an annoying sense that causes an unpleasant experience [1, 2].

The pain perception and its sensation differentiate depending on how interpreted by different individuals in response to a stimulus, so it differs according to age, gender, psychological condition, and cultural aspects [3]. The pain sensation is related to other orthodontic procedures such as the placement of separating elastics, insertion of arch-wires and their subsequent activation, bracket removal, as well as the effects of orthopedic forces [4]. The pain sensation related to orthodontic procedures reaches its peak after 24 h and tends to decline after four days [5].

To control discomfort and pain during orthodontic treatment, different pharmacological and non-pharmacological methods were used [6], such as photobiomodulation (low-level laser, LED) [7, 8], and low-intensity pulsed ultrasound (LIPUS) treatment [9].

Because of its anti-inflammatory properties and regenerative effect on neurons, low-level laser therapy (LLLT) was used to control pain [4, 7]. These effects were attributed to the photobioactive reaction that stimulates the proliferation and differentiation of cells [10].

Several studies showed that LLLT reduced orthodontic pain [4, 7, 11]. While in contrast, other studies showed no significant pain reduction occurred with lasers compared to a placebo [8, 12, 13]. However, it is hypothesized that a more frequent application of laser therapy during the pain/discomfort period might lead to a more recognized reduction in the perception of pain in orthodontic patients [8].

Ultrasound was widely used in medicine as a therapeutic, operative, and diagnostic tool, and recently for pain management [9, 14]. It is generally accepted that LIPUS has no harmful, carcinogenic, or thermal effects to produce biological changes in living tissues [15, 16]. Regarding the LIPUS in reducing pain, only two trials assessed its impact on orthodontic pain [9, 17]. One of them assessed the effect of LIPUS on reducing pain related to canine retraction [9], the other one evaluated its effect of minimizing the pain induced by orthodontic separation [17]. The LIPUS was applied extra-orally in both trials.

Because of its easy use, the ultrasound toothbrush had more interest recently. Some trials evaluated its role in enhancing the health of the periodontal tissues during the orthodontic treatment [18–21]. Since the 1960s, the electric brushes have been modified. More recently, ultrasonic toothbrushes were introduced. Even though the results of various studies have not shown conclusive evidence for ultrasonic brushing effectiveness, the technology might have many advantages compared to other manual and extraoral devices. As the facility of its use encourage us to expert its effectiveness in reducing the orthodontic pain, which many patients face it during the orthodontic treatment, and may prevent some of them from completing their treatment. On the other hand, the high cost of the ultrasonic toothbrushes is one of their disadvantages [19].

According to our knowledge, no studies assessed the impact of LIPUS as a toothbrush in reducing separation pain. In addition, the comparison between the effectiveness of LLLT and LIPUS in reducing orthodontic pain was not also evaluated. Therefore, the purpose of the current study is to evaluate the effectiveness of LLLT and LIPUS in reducing pain induced by orthodontic separation and to compare them. The null hypothesis was that the application of the LLLT or the LIPUS toothbrush does not reduce the separation pain.

## Methods

### Trial design

**Fig. 1:**
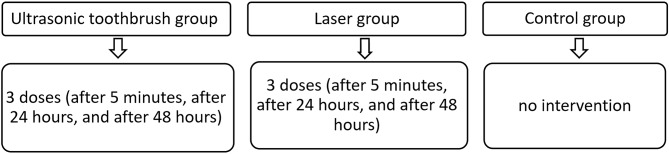
Research design flow chart

The Consolidated Standards of Reporting Trials (CONSORT) checklist was used as a guideline for conducting and reporting this trial [22]. This trial was a three-arm clinical randomized controlled trial; the design was clarified in Fig. 1. It was approved by the Local Ethics Committee of the University of Damascus, Dental School, Syria (UDDS-708-01092020/SRC-3286), and was registered with the German Clinical Trials Register (DRKS). (https://www.drks.de/drks_web/navigate.do?navigationId=trial.HTML&TRIAL_ID= DRKS00029991).

### Participants

Patients were recruited between August 2022 and February 2023 in the Department of Orthodontics, Damascus University.

### Inclusion criteria

Patients aged between 18 and 25 years with complete eruption of all four first permanent molars were included. Tight interproximal contacts points to adjacent second premolar and second permanent molar must be found with no caries and restorations on posterior teeth.

### Exclusion criteria

Patients with previous orthodontic treatment; severely rotated first or second molars; metabolic and periodontal diseases or medications were excluded.

### Clinical interventions

**Fig. 2:**
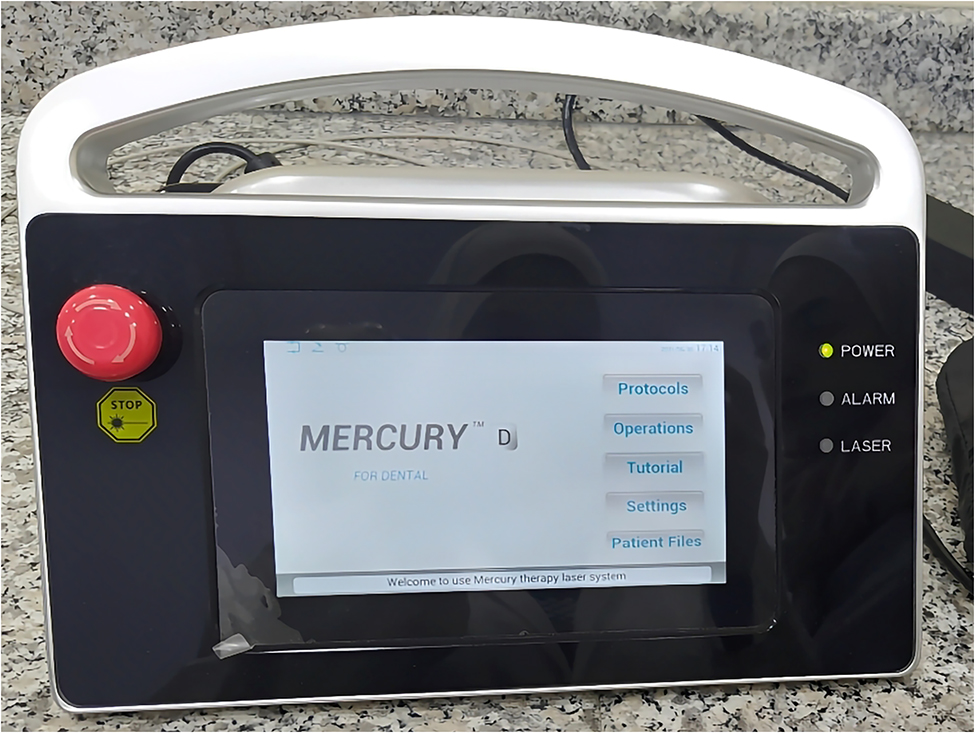
LLLT device

After 5 min of 2.1 mm elastomeric separators placement (Dentalastics Separators, Dentaurum, Ispringen, Germany), subjects of group A underwent low-level laser therapy using soft tissue diode LASER (Mercury-Dual, Pioon Medical Laser, China) (Fig. 2). They were exposed at 8 points (cervical and apical) on the buccal and lingual mucosa (mesially and distally).

**Fig. 3:**
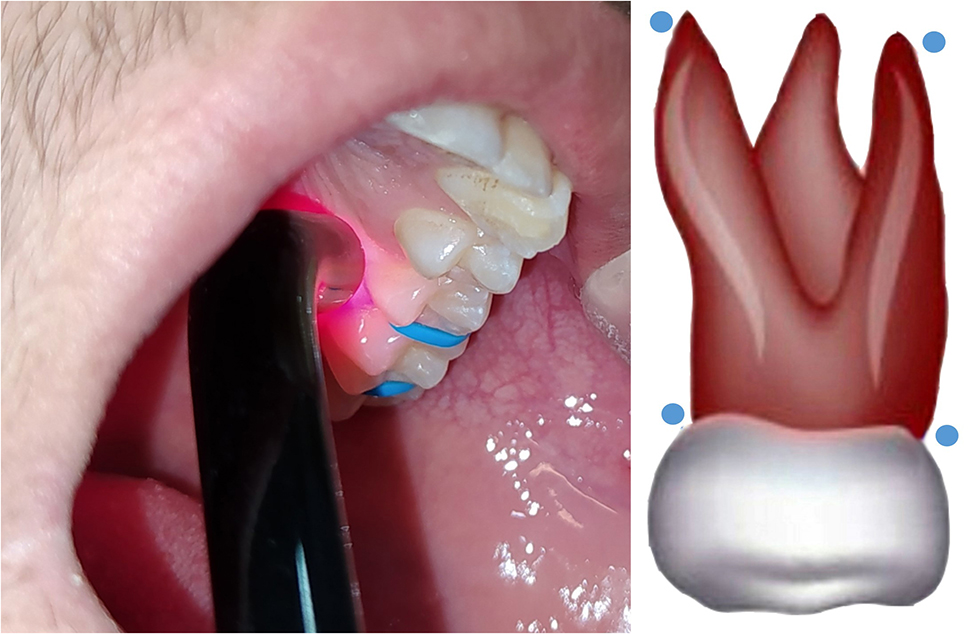
Application of the LLLT on the first molars

The exposure was for 20 s at each point, with an 810-nm aluminum-gallium-arsenide (AlGaAs) diode laser on continuous mode, and output power set at 150 mW, an energy density of 4 J/cm^2^, and a laser spot diameter of 7 mm. Which was on both maxillary and mandibular first molars. The application was repeated after 24, 48 h with close contact between the laser device tip and mucosa (Fig. 3).

**Fig. 4:**
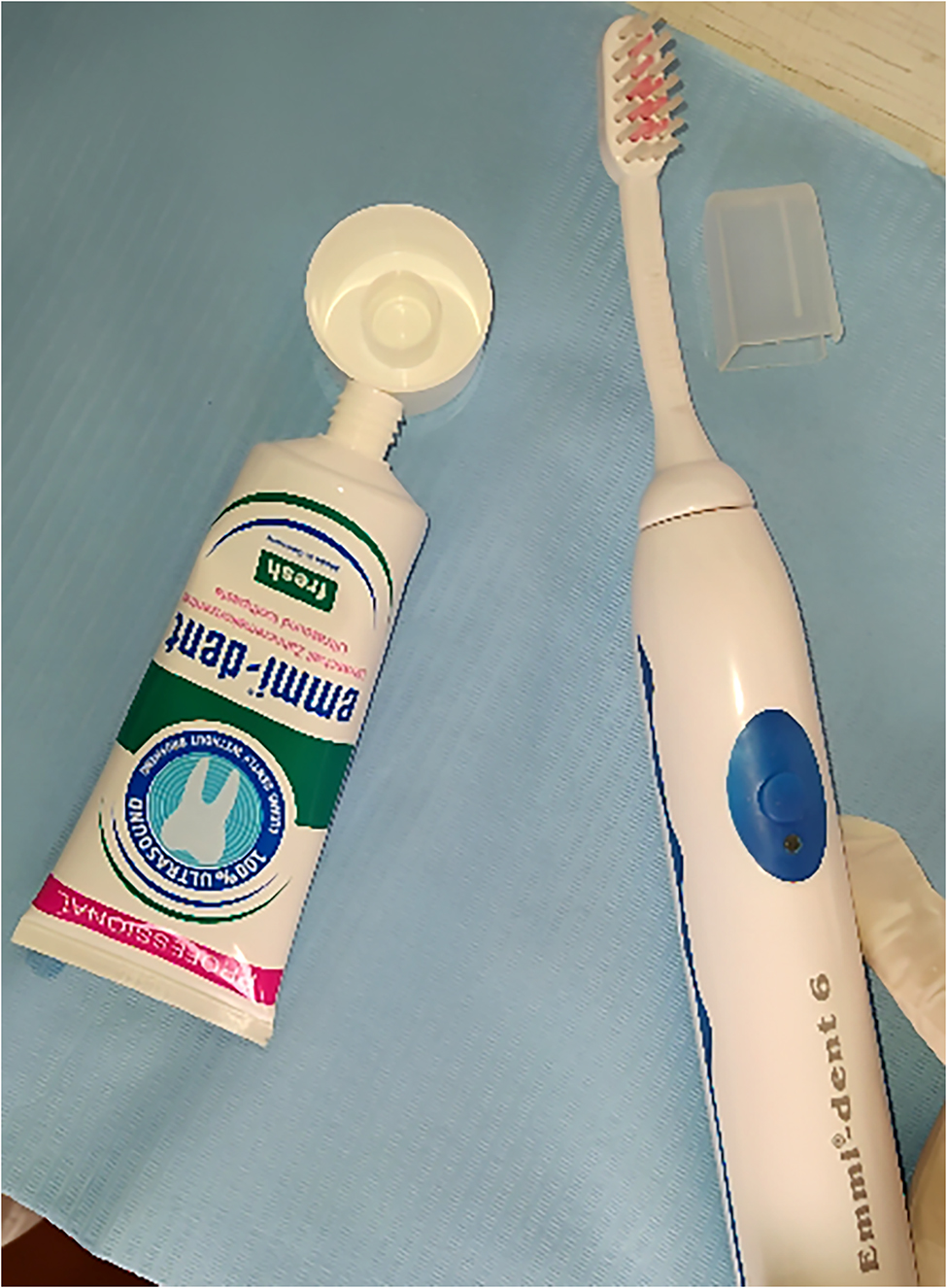
LIPUS toothbrush

Subjects in group B were exposed to the ultrasound toothbrush (Emmi Ultrasonic Co, Morfelden-Waldorf, Germany) (Fig. 4), which generates a frequency of 1.6 MHz; and average output intensity of 0.2 W/cm^2^. The application was for 20 min (5 min on each first molar) after 5 min from separators’ placement, then after 24 and 48 h with no motivation (according to the manufacturer company information).

**Fig. 5:**
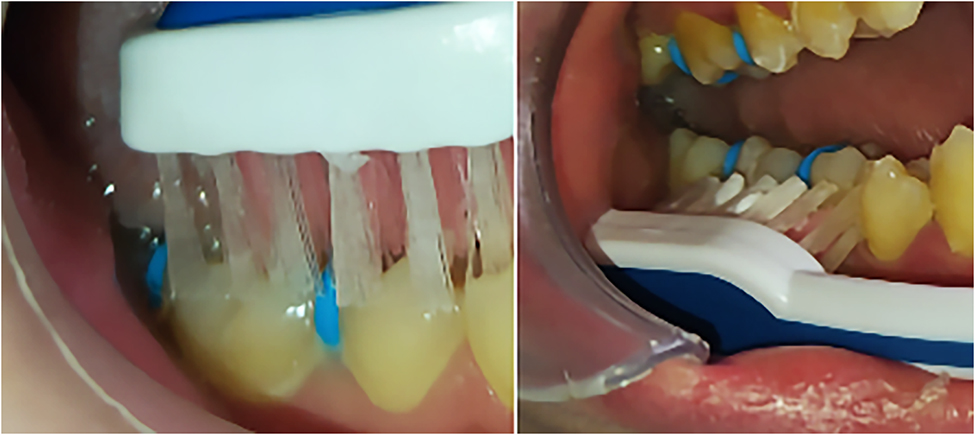
Application of the LIPUS on the first molars

The toothbrush was placed with an angle of 90 degrees to the gingiva for 4 min, then placed vertically on the occlusion surface for 1 min (for each molar) (Fig. 5). Subjects in group C were treated as controls without another intervention after the separators’ placement.

### Outcomes assessment (pain assessment)

The three groups were asked to mark pain severity on a questionnaire containing eight copies of a 100 mm visual analog scale [23] at 5 min, 1, 6, 12, 24, 48, 72, and 96 h after the separators’ placement.

### Sample size calculation

The sample size was calculated using G-Power software (Version 3.0.10 Universitate Keil, Germany), assuming that the effect size of the variable (pain) is 0.549, depending on Kim et al. 2013 (8). When paired t-test with a significance level of 5% and a power of 95% was employed, 138 patients were required to assign randomly into three groups: LLLT group (*n* = 46), LIPUS group (*n* = 46), and control group (*n* = 46). A 10% attrition percentage was added to compensate for any possible attrition. The total sample size was 150 (50 patients per group).

### Randomization, allocation concealment, and blinding

Computer-generated randomization lists with an allocation ratio of 1:1:1 were conducted by an academic staff member (not involved in this research) using Minitab®, v. 17 (Minitab Inc., State College, USA). The allocation sequence was concealed using sequentially numbered, opaque, sealed envelopes which were opened only when the separators were placed. Blinding of personnel and participants were not applicable. Therefore, blinding was applied only for the outcomes’ assessor.

### Statistical analysis

All the statistical analysis was conducted using software program Statistical Package for Social Sciences (IBM SPSS Data Editor Version 24, USA). The Kolmogorov-Smirnov test was used to assess the distribution of data. Data were not normally distributed; thus, the non-parametric tests were applied. Kruskal-Wallis was used to compare the three groups, and post-hoc Mann-Whitney U test was applied to compare each two groups. Friedman test was used to compare the time intervals in each group, followed by Wilcoxon test. Bonferroni correction was applied to compensate for multiple comparison. The significance level was set at 0.05. One author (XXX), who was blinded to all measurements, performed the statistical analysis.

## Results

One hundred and seventy patients were assessed in this trial. Out of them, 150 patients met the inclusion criteria and assigned to the three groups (50 patients per group). Two patients lost to follow-up due to personal reasons in the laser group. One patient did not receive the allocated intervention in the ultrasound group, and another patient was excluded due to irregularities in his VAS chart completion. One patient did not come to the determined appointments in the control group. Consequently, one hundred and forty-five patients (70 males and 75 females aged from 18 to 25 years) were enrolled in this study.

**Fig. 6:**
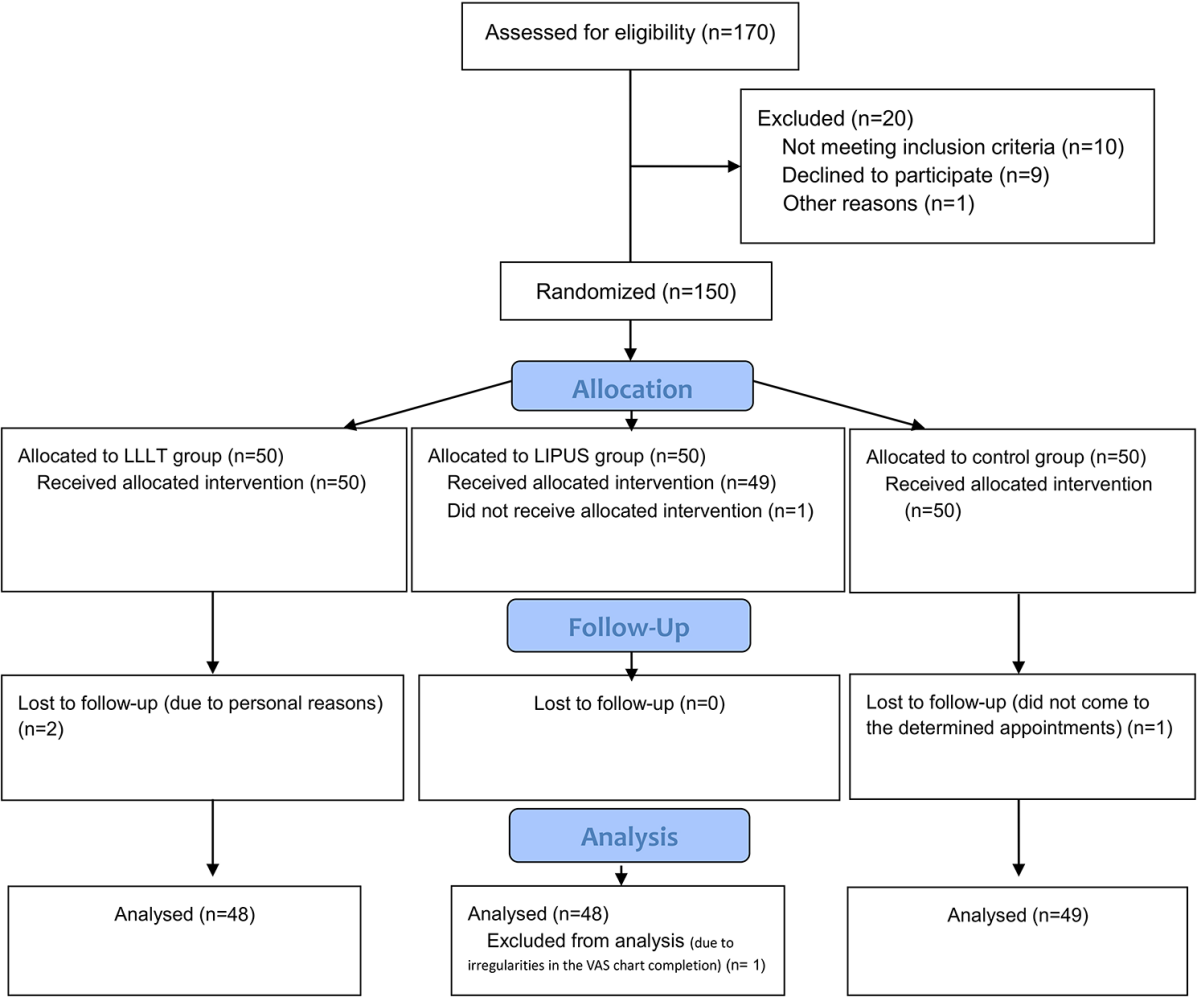
CONSORT flow diagram

Patients’ allocation and follow-up are given in Fig. 6. The basic characteristics of the sample are shown in Table 1. The follow-up duration was four days.

**Table 1:** Baseline characteristics of the three groups

	LLLT group	LIPUS group	Control group	*P*-Value	Significance
N Age Y Gender; Female Male	48 21.52 ± 2.20 28 20	48 21.16 ± 2.16 27 21	49 21.44 ± 2.16 20 29	0.823 0.304	NS NS

n, number of subjects; Y, year; NS, Non-Significant

There were no significant differences in age and gender among the laser, ultrasound, and control groups (*P* = .823, 0.304, respectively) (Table 1). There was a significant difference in pain perception among the three groups after 5 min (pain scores were as following: LLLT 4.16, LIPUS 4.06, and Control 16.45) (*P* = .002) (Table 2). Both the laser group and the ultrasound group showed a statistically significant decrease in pain scores compared to the control group (*P* < .001), with no differences between the laser group and the ultrasound group in reducing the pain scores (*P* > .05) (Table 3). On the other hand, gender had no significant effect on pain perception scores at all the assessment points (*P* > .05).

**Table 2:** Means and standard deviations of pain scores in the LLLT and LIPUS and control groups at different time points

	5 min	1 h	6 h	12 h	24 h	48 h	72 h	96 h
LLLT group LIPUS group Control group *P*-Value ^a^	4.16 ± 6.21 4.06 ± 5.89 16.45 ± 21.9 0.002	2.18 ± 4.82 2.60 ± 4.72 19.79 ± 23.08 < 0.001	4.68 ± 4.99 6.97 ± 6.82 38.75 ± 28.27 < 0.001	6.35 ± 6.66 4.68 ± 5.68 51.66 ± 31.52 < 0.001	12.70 ± 9.50 11.35 ± 9.60 57.91 ± 27.20 < 0.001	2.18 ± 4.60 2.50 ± 5.25 47.91 ± 25.17 < 0.001	0.72 ± 2.30 1.04 ± 3.56 39.79 ± 25.45 < 0.001	2.50 ± 3.71 3.12 ± 4.07 31.04 ± 23.01 < 0.001

^a^Kruskal Wallis test; C indicates control group; LLLT, low-level laser group; LIPUS, low-intensity pulsed ultrasound group

The maximum pain level was reached after 24 h of the separators’ placement in the three groups. The laser group had a mean score of (12.70 ± 9.50), whereas the mean score in the ultrasound group was (11.35 ± 9.60), which was significantly lower than that in the control group (57.91 ± 27.20). The pain scores of the three groups decreased from the second to the fourth day after the separators’ placement. In all groups, there were significant differences between pain scores at most different time points (Supplementary Tables 1, 2, 3).

**Table 3:** The multiple comparisons between the LLLT, the LIPUS, and the control groups at different time points

Evaluation time	Multiple comparisons ^a^		
	LLLT-C	LIPUS-C	LLLT-LIPUS
5 min 1 h 6 h 12 h 24 h 48 h 72 h 96 h	(*P* = .002) (*P* < .001) (*P* < .001) (*P* < .001) (*P* < .001) (*P* < .001) (*P* < .001) (*P* < .001)	(*P* = .003) (*P* < .001) (*P* < .001) (*P* < .001) (*P* < .001) (*P* < .001) (*P* < .001) (*P* < .001)	(*P* = .89) NS (*P* = .49) NS (*P* = .12) NS (*P* = .21) NS (*P* = .33) NS (*P* = .94) NS (*P* = .96) NS (*P* = .45) NS

^a^mann-whitney U test; C indicates control group; LLLT, low-level laser group; LIPUS, low-intensity pulsed ultrasound group; NS, non-significant

## Discussion

This trial aimed to evaluate and compare the effect of multiple applications of LLLT and LIPUS on reducing the pain associated with the placement of orthodontic separators. The laser used in this trial was the soft tissue diode laser (AlGaAs), with a wavelength of 810 nm. The energy dose of 1.5 W was applied at eight buccal and lingual points. The application was performed for three doses. Regarding the results of the current trial, the null hypothesis was rejected.

There was a significant decrease in the pain scores at all the assessment time points when the LLLT was applied compared to the control group, which was in agreement with many previous studies [7, 11, 24–28]. Otherwise, Kim et al. found that the LLLT reduces the separation pain only after the first day from placement of separators when compared with placebo and control groups [8]. Furthermore, Abtahi et al. [12], AlSayed Hasan et al. [13] and Furquim et al. [29] found no significant difference between the laser group and the placebo, which might be explained by the fact that the application of laser was different (type of the laser, wavelength, doses, and the placement of application). Moreover, another limitation observed in one of the previous studies was that the method used in the sample calculation was unclear [12].

Laser irradiation has a variety of effects on tissues, and that depends on the wavelength of laser. The beneficial action of laser irradiation is the result of free radical reactions including activation of cells (leucocytes, fibroblasts, and keratinocytes) which is expressed in production of protein and cytokines, and cell proliferation. All these events are the basic of the therapeutic action of laser therapy [30–33]. However, the low-level laser therapy may have a placebo effect on the pain related to orthodontic procedures, which needs more studies to detect it.

Ultrasound was used in this trial as a toothbrush (Emmi Ultrasonic Co, Morfelden-Waldorf, Germany, which generates a frequency of 1.6 megahertz and an average output intensity of 0.2 W/centemetre^2^) to verify its effectiveness in controlling orthodontic separation pain. According to our knowledge, there were no studies assessed the effect of this toothbrush on this topic.

The results showed that the pain levels were significantly lower than those in the control group. Regarding the comparison of pain management between the LLLT group and the LIPUS group, the pain scores were similar in both groups with no significant differences.

Concerning the previous studies, there were only two trials assessed the effect of LIPUS on reducing orthodontic pain [9, 17]. However, these two trials applied the LIPUS extra-orally. One trial evaluated the pain perception after placement of separators and found that the ultrasound decreased the pain significantly [17]. The other trial assessed the pain perception during canine retraction and applied it extra orally over the length of the maxillary canine root [9]. The results of this trial showed that the LIPUS was not effective in reducing pain. This different result might be explained by the different pain perceptions of the canine retraction compared to the molar separation.

Regarding the ultrasonic toothbrush mechanism, the manufacturer suggests that the sonic waves (because they are transmitted subgingivally for about 12 mm) might have an effect on the substances that existence in the periodontal area.

The patients reported the maximum pain level after 24 h of separator placement in the three groups. This agrees with the results of many previous studies [24, 34–36], which linked that with the peak level of prostaglandins. There was a remarkable decline in the severity of pain in all groups on days 2 and 3 after the separation, the probable reason that the period of 2 to 4 days is considered critical after the orthodontic procedure on the inflammatory process of the periodontal ligament [37].

Despite the good effect of low-level laser therapy and ultrasound toothbrush on relieving the separation pain, the high cost of both procedures may be an obstacle for some patients. Although the low-level laser is not cost effective, the beneficial effects of multiple applications and the pain relief induced explain the importance of this procedure.

According to the current study, both laser and ultrasound can effectively control the intensity of separation pain. However, this is the first study performed to compare the LLLT and LIPUS toothbrushes in reducing separation pain. Moreover, assessment of the effect of the LIPUS on minimizing the pain associated with different stages of orthodontic treatment and comparison with LLLT efficacy is needed.

### Limitations and generalizability

Despite the rigorous procedures related to sample construction, applied interferences, and data collection, the study findings generalizability are still limited because the pain sensation is a personal experience, so it could not be determined accurately by all participants. Another limitation is that bringing patients into the practice for three consecutive days for the application might not be welcomed by the participants; however the application of LIPUS toothbrush can be applied by the patient himself/herself at home. Nevertheless, the current trial findings might be considered representative to an acceptable extent.

## Conclusions

The multiple doses of the LLLT and the LIPUS were effective in reducing the pain induced by orthodontic separation, and there was no difference between them in the separation pain control.

### Electronic supplementary material

Below is the link to the electronic supplementary material.

Supplementary Material 1

Supplementary Material 2

Supplementary Material 3

## Data Availability

The data used and analysed during the current trial is available from the corresponding author on reasonable request.
