# Supplementary material for: The effectiveness of low-level laser therapy and low-intensity pulsed ultrasound in reducing pain induced by orthodontic separation: a randomized controlled trial
Source: BMC Oral Health. 2024 Feb 2;24:166. doi: 10.1186/s12903-024-03926-2 (PMC10835832; doi:10.1186/s12903-024-03926-2)
Supplement: Supplementary file 1 — Supplementary Material 1 [file 12903_2024_3926_MOESM1_ESM.docx]

**Supplementary Table 3:** Differences between the interval time points in the Control group ^a^.

|  | 5 m | 1 h | 6 h | 12 h | 24 h | 48 h | 72 h | 96 h |
| --- | --- | --- | --- | --- | --- | --- | --- | --- |
| 5 m  1 h  6 h  12 h  24 h  48 h  72 h  96 h | -  .307  .000  .000  .000  .000  .000  .003 | .307  -  .000  .000  .000  .000  .000  .009 | .000  .000  -  .000  .000  .074  .990  .058 | .000  .000  .000  -  .016  .416  .028  .000 | .000  .000  .000  .016  -  .002  .000  .000 | .000  .000  .074  .416  .002  -  .005  .000 | .000  .000  .990  .028  .000  .005  -  .001 | .003  .009  .58  .000  .000  .000  .001  - |

^a^  Wilcoxon test.
