# Supplementary material for: The effectiveness of low-level laser therapy and low-intensity pulsed ultrasound in reducing pain induced by orthodontic separation: a randomized controlled trial
Source: BMC Oral Health. 2024 Feb 2;24:166. doi: 10.1186/s12903-024-03926-2 (PMC10835832; doi:10.1186/s12903-024-03926-2)
Supplement: Supplementary file 2 — Supplementary Material 2 [file 12903_2024_3926_MOESM2_ESM.docx]

**Supplementary Table 2:** Differences between the interval time points in the LIPUS group ^a^.

|  | 5 m | 1 h | 6 h | 12 h | 24 h | 48 h | 72 h | 96 h |
| --- | --- | --- | --- | --- | --- | --- | --- | --- |
| 5 m  1 h  6 h  12 h  24 h  48 h  72 h  96 h | -  .111  .020  .920  .000  .144  .002  .358 | .111  -  .000  .257  .000  .898  .048  .301 | .020  .000  -  .002  .237  .000  .000  .001 | .920  .257  .002  -  .005  .155  .002  .541 | .031  .001  .524  .013  -  .004  .000  .004 | .141  .898  .000  .155  .000  -  .042  .441 | .002  .048  .000  .002  .000  .042  -  .002 | .358  .301  .001  .541  .000  .441  .002  - |

^a^  Wilcoxon test.
