# Supplementary material for: The effectiveness of low-level laser therapy and low-intensity pulsed ultrasound in reducing pain induced by orthodontic separation: a randomized controlled trial
Source: BMC Oral Health. 2024 Feb 2;24:166. doi: 10.1186/s12903-024-03926-2 (PMC10835832; doi:10.1186/s12903-024-03926-2)
Supplement: Supplementary file 3 — Supplementary Material 3 [file 12903_2024_3926_MOESM3_ESM.docx]

**Supplementary Table 1**: Differences between the interval time points in the LLLT group ^a^.

|  | 5 m | 1 h | 6 h | 12 h | 24 h | 48 h | 72 h | 96 h |
| --- | --- | --- | --- | --- | --- | --- | --- | --- |
| 5 m  1 h  6 h  12 h  24 h  48 h  72 h  96 h | -  .032  .038  .583  .000  .032  .001  .078 | .032  -  .000  .006  .000  .942  .054  .666 | .038  .000  -  .052  .237  .000  .000  .001 | .583  .006  .052  -  .005  .005  .000  .015 | .000  .000  .237  .005  -  .000  .000  .000 | .032  .942  .000  .005  .000  -  .041  .438 | .001  .054  .000  .000  .000  .041  -  .015 | 0.78  .666  .001  .015  .000  .438  .015  - |

^a^  Wilcoxon test.
